# Comparison of continuous epidural analgesia, traditional combined spinal–epidural, and modified combined spinal–epidural for labor analgesia: a multicenter retrospective cohort study

**DOI:** 10.3389/fmed.2026.1805555

**Published:** 2026-07-08

**Authors:** Xianghuan Chen, Yulu Jin, Min Yu, Miaomiao Yang, Jiehao Sun, Yongliang Li

**Affiliations:** 1 Department of Anesthesiology and Operation, Cangnan Hospital of Wenzhou Medical University (The People’s Hospital of Cangnan), Zhejiang, China; 2 Department of Anesthesiology, Yueqing Third People’s Hospital, Zhejiang, China; 3 Department of Anesthesiology, Ruian Maternal and Child Health Hospital, Zhejiang, China; 4 Department of Anesthesiology, Ruian People’s Hospital, Zhejiang, China; 5 Department of Anesthesiology, The First Affiliated Hospital of Wenzhou Medical University, Zhejiang, China; 6 Department of Anesthesiology, Ruian People’s Hospital (The Third Affiliated Hospital of Wenzhou Medical University), Zhejiang, China

**Keywords:** combined spinal–epidural, epidural analgesia, labor analgesia, maternal outcomes, modified CSE

## Abstract

**Background:**

Neuraxial analgesia is the most effective method for labor pain relief. Continuous epidural analgesia (CEA) provides reliable analgesia but is associated with delayed onset, while traditional combined spinal–epidural analgesia (CSE) offers rapid pain relief at the expense of increased maternal side effects. Modified CSE techniques, typically involving reduced intrathecal drug doses and/or optimized epidural maintenance strategies have been introduced to balance rapid onset with improved safety, but comparative real-world evidence remains limited.

**Methods:**

In this multicenter retrospective cohort study, 120 term parturients received labor analgesia with continuous epidural analgesia (CEA, *n* = 40), traditional combined spinal–epidural analgesia (CSE, *n* = 40), or modified CSE (*n* = 40) at three tertiary hospitals. All intrathecal administrations of bupivacaine were hyperbaric. The primary outcomes were early analgesic efficacy, including time to effective analgesia [visual analog scale (VAS) ≤ 3] and VAS scores within the first 60 min. Secondary outcomes included inadequate analgesia at 1 h (VAS > 4), maternal hemodynamic effects, motor block, labor and delivery outcomes, neonatal outcomes, and maternal satisfaction

**Results:**

Median onset of effective analgesia was significantly shorter with traditional CSE (median difference vs. CEA:-12 min) and modified CSE (median difference vs. CEA:-9 min) compared with CEA (18 min; *p* < 0.001). Inadequate analgesia at 1 h occurred more frequently with CEA (18.1%) than with traditional CSE (4.2%) or modified CSE (6.7%) (absolute risk reduction 11.4% compared with modified CSE). Rescue analgesic interventions during the first hour after neuraxial initiation were comparable across groups (CEA 6.7%, traditional CSE 6.7%, modified CSE 8.3%). Traditional CSE was associated with higher rates of hypotension and motor block, whereas modified CSE demonstrated a safety profile comparable to CEA. Maternal hemodynamic stability, labor duration, delivery mode, and neonatal outcomes were similar among the three groups. However, the study was underpowered to detect differences in rare maternal or neonatal adverse events, and these findings should be interpreted cautiously. Maternal satisfaction was highest in the modified CSE group.

**Conclusion:**

Modified CSE provides faster early analgesia than CEA and is associated with fewer episodes of pain exceeding a VAS score of 4 a while requiring similar rates of rescue analgesic interventions as CEA and traditional CSE, and maintaining better hemodynamic stability and less motor block than traditional CSE. All intrathecal bupivacaine doses were hyperbaric. Other maternal and neonatal outcomes should be interpreted cautiously due to limited power for rare events. Overall, the results support modified CSE as a pragmatic refinement of neuraxial labor analgesia in routine clinical practice.

## Introduction

Labor pain is one of the most intense forms of pain experienced by women and can have significant physiological and psychological consequences if inadequately managed. Inadequate labor analgesia has been associated with adverse maternal stress responses and negative childbirth experiences, which may influence overall satisfaction with delivery (1, 2). Therefore, ensuring effective and timely pain relief is a central goal of modern obstetric care.

Neuraxial analgesia is widely regarded as the most effective and reliable method for labor pain relief. Among neuraxial techniques, CEA and CSE are the most frequently used approaches worldwide (3, 4). CEA provides flexible, titratable, and sustained analgesia throughout labor and is associated with high maternal satisfaction. However, its slower onset of action and the risk of inadequate early pain control remain clinically relevant limitations, particularly during the active phase of labor (4, 5).

Traditional CSE was introduced to overcome the slower onset associated with epidural-only techniques. By combining an intrathecal injection with epidural catheter placement, CSE offers rapid onset and dense initial analgesia, often resulting in superior pain relief in the early stages of labor (6). This benefit, however, comes with a slightly higher risk of post-dural puncture headache compared with epidural-only techniques, although overall rates remain low when small-gauge pencil-point needles are used to minimize this risk (7). This benefit, however, comes at the cost of increased maternal side effects, including hypotension, pruritus, and fetal heart rate changes, as well as challenges in maintaining stable analgesia once the spinal effect diminishes (8, 9). These trade-offs have prompted ongoing efforts to refine neuraxial techniques that can preserve rapid onset while improving maternal safety and stability.

In recent years, modified CSE techniques have emerged in clinical practice, most commonly involving reduced intrathecal drug doses, altered drug composition, or optimized timing and dosing of epidural maintenance (3, 10, 11). From a mechanistic perspective, these modifications aim to limit sympathetic blockade and motor impairment while retaining the rapid sensory analgesia achieved through intrathecal drug delivery. Despite increasing adoption, comparative evidence evaluating modified CSE against both conventional CEA and traditional CSE remains limited, especially in multicenter, real-world settings where variations in technique and patient characteristics are common.

Early analgesic performance is of particular clinical importance in labor analgesia. Insufficient pain relief during the first hour after neuraxial initiation is strongly associated with maternal distress, increased need for rescue interventions, and reduced satisfaction with the childbirth experience (12). However, most prior studies have focused on overall analgesic efficacy rather than clinically meaningful early endpoints or the incidence of inadequate analgesia. Therefore, we conducted a multicenter retrospective cohort study to test the hypothesis that modified CSE provides more rapid achievement of effective analgesia and is associated with fewer parturients experiencing clinically significant pain (VAS > 4) and fewer requiring rescue analgesic interventions during the first hour after neuraxial initiation, compared with both CEA and traditional CSE, while maintaining a safer maternal side-effect profile. Specifically, we compared early onset time, pain scores within the first 60 minutes, the incidence of pain exceeding a VAS score of 4 and the need for rescue analgesia, and maternal and neonatal outcomes under routine clinical conditions, with the aim of informing evidence-based selection of neuraxial labor analgesia techniques in contemporary practice.

## Materials and methods

### Study design and setting

This study was conducted as a multicenter retrospective cohort investigation at three tertiary referral hospitals providing comprehensive obstetric and anesthesia services. The study period extended from May 30, 2025, to October 30, 2025. Clinical data were obtained from electronic medical records and anesthesia information systems at each participating institution. Multicenter retrospective designs are commonly used in obstetric anesthesia research to evaluate real-world practice. This design acknowledges inherent methodological strengths and limitations (13, 14).

All neuraxial labor analgesia procedures were performed as part of routine clinical practice. All intrathecal bupivacaine administered was hyperbaric. No additional interventions were required for the purposes of this study. A retrospective cohort design was selected to evaluate real-world clinical practice across multiple centers. This design did not interfere with standard obstetric or anesthetic management, in accordance with prior observational research in labor neuraxial analgesia (13, 14). Given the multicenter design, potential center-level differences in clinical practice were anticipated. Therefore, center was considered in exploratory sensitivity analyses to assess the robustness of the primary outcomes across institutions. Although some aspects of data collection, such as the timing of pain assessments and maternal satisfaction measurements, followed routine clinical protocols established at the participating centers, the study adhered to a retrospective design. These elements were part of the standard clinical care protocol, which was implemented as part of the routine obstetric practice at the hospitals, not as a prospective research intervention.

### Participants

#### Inclusion and exclusion criteria

Parturients were eligible for inclusion if they met the following criteria: singleton pregnancy at term (gestational age ≥ 37 weeks), cephalic fetal presentation, planned or attempted vaginal delivery, and receipt of neuraxial labor analgesia using one of the following techniques: continuous epidural analgesia (CEA), traditional CSE, or modified CSE. Only cases with complete clinical, anesthesia, and outcome data for the predefined study endpoints were included.

**FIGURE 1:**
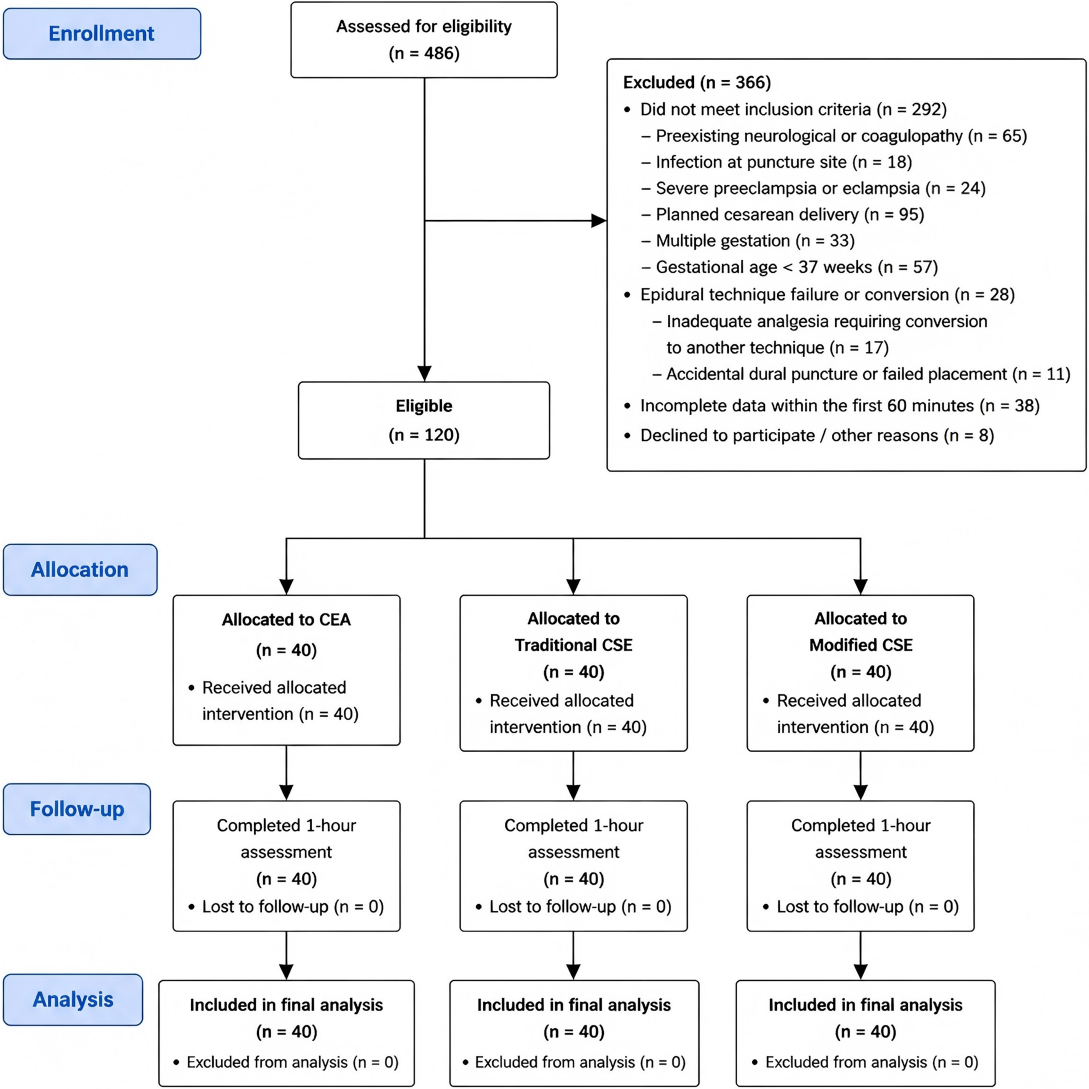
Flow of participants through the study.

Exclusion criteria included contraindications to neuraxial anesthesia, such as coagulopathy or infection at the puncture site. Other exclusion criteria included severe maternal comorbidities likely to affect hemodynamic stability or labor progression, including severe preeclampsia or structural heart disease. Additional exclusions were multiple gestation, non-cephalic presentation, and incomplete or missing data related to analgesia technique, pain assessment, or key maternal or neonatal outcomes. The most frequently missing variables were repeated early VAS measurements and maternal satisfaction scores. Baseline demographic and obstetric characteristics of excluded parturients were reviewed. These characteristics did not differ meaningfully from those of included participants, which reduces the likelihood of systematic exclusion bias. The final analysis included 120 parturients, with 40 in each group (CEA, traditional CSE, and modified CSE). Detailed participant flow and reasons for exclusion are presented in Figure 1.

#### Group classification

Participants were assigned to one of three groups based on the neuraxial labor analgesia technique actually used in routine clinical care, as documented in anesthesia records: the CEA group, the traditional CSE group, or the modified CSE group. A total of 120 parturients were included in the final analysis, with 40 parturients in each group (CEA, traditional CSE, and modified CSE).

Within the analytic framework of the study, CEA was considered the conventional comparator. Modified CSE was designated as the investigational technique, while traditional CSE served as an active (positive) comparator due to its established association with rapid onset and effective early labor analgesia. Because technique selection was determined by anesthesiologist judgment and patient preference rather than randomization, confounding by indication was anticipated. This was considered in the interpretation of comparative outcomes. Where feasible, clinician- and center-related factors were included in adjusted analyses.

#### Neuraxial analgesia techniques

All neuraxial procedures were performed by experienced anesthesiologists in accordance with contemporary standards of labor analgesia practice (15).

For continuous epidural analgesia, the procedure was initiated with the parturient in either the sitting or lateral decubitus position. Epidural puncture was performed at a lumbar interspace, most commonly L2–L3 or L3–L4, using a standard loss-of-resistance technique. An epidural catheter was threaded 3–5 cm into the epidural space. Following catheter placement, a loading dose of low-concentration local anesthetic (e.g., ropivacaine 0.1–0.125%, 10–15 mL) with or without an opioid adjuvant was administered. Analgesia was maintained using continuous epidural infusion and/or patient-controlled epidural analgesia (PCEA), initiated immediately after the loading dose, with maintenance regimens and concentrations adjusted according to institutional protocol.

Traditional CSE was performed using a needle-through-needle technique at a lumbar interspace. After identification of the epidural space, a spinal needle was advanced into the subarachnoid space, and correct placement was confirmed by free flow of cerebrospinal fluid. An intrathecal dose of hyperbaric bupivacaine (e.g., 2.5 mg) with or without fentanyl (15–25 μg) was administered, followed by insertion of an epidural catheter for maintenance of labor analgesia. Continuous epidural infusion or PCEA was initiated immediately after intrathecal injection according to center-specific protocols (6, 11).

Modified CSE: Modified CSE was operationally defined as a combined spinal–epidural technique using reduced intrathecal local anesthetic doses of hyperbaric bupivacaine (1.25–2.5 mg) with or without fentanyl (10–25 μg), delayed or modified timing of epidural activation, and/or lower-concentration epidural maintenance regimens (e.g., ropivacaine 0.0625–0.1% with fentanyl 1–2 μg/mL), as determined by institutional protocol. These variations are summarized in Supplementary Tables 1, 2, which provide comprehensive center-specific protocol details for all three techniques. The term “modified CSE” in this study refers to a class of technique refinements rather than a single standardized protocol.

#### Sample size and power analysis

The sample size for this study was calculated based on the primary outcome of early analgesic efficacy (time to effective analgesia). Assuming a moderate effect size (Cohen’s *d* = 0.5) between the groups for the primary outcome, a power of 80%, and an alpha level of 0.05, a minimum of 40 participants per group was required. This sample size was calculated using [name of statistical software] for an independent samples *t*-test. It was determined that this sample size would provide adequate power to detect clinically meaningful differences in the primary outcomes.

For secondary outcomes, such as neonatal outcomes and rare adverse events, the study may be underpowered due to the relatively small sample size. A post hoc power analysis was conducted to assess the risk of Type II errors for these outcomes. The analysis indicated that the power to detect differences for neonatal outcomes and rare adverse events was approximately 56 and 45%, respectively. This is inadequate given the sample size.

#### Monitoring and clinical management

Maternal monitoring included regular measurements of noninvasive blood pressure, heart rate, and oxygen saturation (SpO2). Continuous fetal heart rate monitoring was performed in accordance with standard obstetric practice. Maternal hypotension was managed with intravenous fluid administration and vasopressor therapy, including phenylephrine or ephedrine, as per institutional protocols. Other adverse effects such as nausea, vomiting, and pruritus were treated as clinically indicated.

Motor block was evaluated using a modified Bromage scale, in which 0 indicates full flexion of the knees and ankles, 1 indicates partial flexion of the knees with full ankle movement, 2 indicates inability to flex the knees with preserved ankle flexion, and 3 represents inability to move either the knees or ankles. Assessments were performed at predefined intervals during the first 30 min after initiation of neuraxial analgesia.

Maternal SpO2 and heart rate were monitored continuously. Noninvasive blood pressure measurements were obtained at frequent intervals during the early phase of analgesia and then at longer intervals thereafter, in accordance with institutional protocols. Hypotension was defined as a systolic blood pressure less than 90 mmHg or a reduction to less than 80% of the pre-analgesia baseline. Episodes of hypotension were treated with rapid administration of crystalloid solution and intravenous vasopressors such as phenylephrine or ephedrine. Bradycardia, defined as a heart rate less than 50 beats per minute, was treated with intravenous atropine 0.5 mg when clinically indicated.

Inadequate analgesia was defined a priori as a visual analogue scale (VAS) score greater than 4 at 1 h after initiation of neuraxial analgesia (5, 16). VAS scores were routinely recorded by bedside nursing staff as part of standard labor documentation. Rescue management strategies included epidural bolus dosing, adjustment of infusion parameters, maternal repositioning, or conversion to an alternative analgesic technique. All rescue interventions and subsequent pain score changes were documented and included in the analysis.

Adverse effects including hypotension, nausea, vomiting, pruritus, and motor block were recorded. Pain intensity was assessed using a 10-cm VAS, where 0 represents no pain and 10 represents the worst pain imaginable. Maternal satisfaction with analgesia was assessed using a VAS ranging from 0 (completely dissatisfied) to 10 (completely satisfied), recorded within 30 min after delivery once the patient had the opportunity to reflect on the entire labor experience. While maternal satisfaction is a multidimensional concept, we used the VAS as a unidimensional, quantitative proxy for overall satisfaction. This standardized approach allowed consistent measurement and comparison across all participants.

### Outcome measures

#### Primary outcomes

The primary outcomes were measures of early analgesic efficacy. These included the onset time of effective analgesia, defined as the interval from completion of neuraxial drug administration of hyperbaric bupivacaine to achievement of a VAS score ≤ 3, and VAS pain scores recorded at baseline and at 5, 10, 20, 30, and 60 min following initiation of analgesia. The focus on early analgesic onset was based on prior evidence demonstrating clinically meaningful differences between combined spinal–epidural and epidural techniques during the initial phase of labor analgesia (17, 18). The principal analytic focus was the comparison between the modified CSE and CEA groups, with traditional CSE serving as a reference comparator.

#### Secondary outcomes

Secondary outcomes included the incidence of inadequate analgesia at 1 h. Other secondary outcomes included the duration of the first and second stages of labor, total labor duration, and maternal hemodynamic changes. The mode of delivery, incidence of maternal adverse effects, and maternal satisfaction (when documented) were also assessed. Neonatal condition was evaluated based on Apgar scores at 1 and 5 min, requirement for neonatal resuscitation, and admission to the neonatal intensive care unit (NICU)

#### Data collection and statistical analysis

Data were extracted from electronic medical records using standardized case-report forms. Variables collected included maternal demographic and obstetric characteristics, details of neuraxial technique and drug regimens, VAS pain scores at predefined time points, rescue analgesia interventions, maternal hemodynamic parameters, labor duration, delivery mode, and neonatal outcomes. Continuous variables were summarized as mean ± standard deviation or median with interquartile range (IQR), depending on the distribution. Categorical variables were presented as counts and percentages. In addition to unadjusted group comparisons, multivariable regression analyses were performed to account for potential confounding variables. These included maternal age, body mass index, parity, cervical dilation at analgesia initiation, and study center. Adjusted effect estimates with corresponding confidence intervals were calculated for primary outcomes. Comparisons among groups were otherwise performed using analysis of variance or Kruskal–Wallis tests for continuous variables and chi-square or Fisher’s exact tests for categorical variables, as appropriate. All tests were two-sided, and a *p* < 0.05 was considered statistically significant. Given that multiple comparisons were made between the three groups, the Bonferroni correction was applied to adjust the significance level to reduce the risk of Type I errors. To assess the impact of protocol variability across centers, we included center-specific data for the modified CSE technique in Supplementary Table 2. This table presents the variability in hyperbaric intrathecal bupivacaine and fentanyl doses used across the participating centers. Sensitivity analyses were conducted to exclude outlier protocols where there were significant deviations from the mean doses of hyperbaric intrathecal bupivacaine and fentanyl, as detailed in Supplementary Table 2. This allowed us to examine whether the findings were influenced by specific centers or outlier data. The results of these sensitivity analyses showed that excluding outlier protocols did not significantly alter the primary findings related to the early analgesic efficacy and maternal safety outcomes.

#### Ethics approval and regulatory considerations

This research protocol for this retrospective study involving human participants was approved by the Institutional Review Board (IRB) at Ruian People’s Hospital (The Third Affiliated Hospital of Wenzhou Medical University) (YJ2025-049-01). Given the retrospective design and use of de-identified routinely collected clinical data, the requirement for written informed consent was waived. All data were handled confidentially, and no patient-identifying information was accessible to the investigators during data analysis.

Informed consent was waived for this retrospective study in accordance with institutional ethical guidelines, as it involved the use of de-identified, routinely collected clinical data. This waiver was approved by the Institutional Review Board at each participating hospital. Given the retrospective nature of the study, patient-identifying information was not accessible to the investigators, ensuring participant confidentiality. Access to the data used in this study may be granted upon reasonable request, subject to institutional approval and compliance with ethical guidelines. The datasets used and/or analyzed during this study are not publicly available due to privacy and ethical restrictions. The data were anonymized to ensure patient confidentiality. However, access to the data may be provided upon reasonable request, subject to institutional and ethical guidelines. Interested parties should submit their request to the respective institutional review boards for approval.

## Results

### Study population

A total of 486 parturients receiving neuraxial labor analgesia were screened. After applying exclusion criteria 366 participants excluded due to not meeting eligibility criteria (*n* = 292), epidural technique failure or conversion (*n* = 28), incomplete data within the first 60 min (*n* = 38), or other reasons such as declining participation (*n* = 8). A total of 120 parturients receiving hyperbaric intrathecal bupivacaine were included in the final analysis. Of these, 40 parturients received CEA, 40 received traditional combined spinal–epidural analgesia (CSE), and 40 received modified CSE. Participant flow through the study is presented in Figure 1. Baseline maternal demographic and obstetric characteristics were similar among the three groups (Table 1). Mean maternal age was approximately 31 years, mean body mass index was 26 kg/m^2^, mean gestational age at delivery was 39 weeks, and approximately half of the parturients in each group were nulliparous. Cervical dilation at the time of neuraxial placement did not differ significantly between groups.

**TABLE 1:** Baseline characteristics of parturients.

Characteristic	CEA	Traditional CSE	Modified CSE	*P*-value
Maternal age (years)	31 ± 4 (95% CI: [30, 32])	31 ± 4 (95% CI: [30, 32])	31 ± 4 (95% CI: [30, 32])	0.91
Body mass index (kg/m^2^)	26 ± 3 (95% CI: [25.5, 26.5])	26 ± 3 (95% CI: [25.5, 26.5])	26 ± 3 (95% CI: [25.5, 26.5])	0.87
Gestational age (weeks)	39 ± 1 (95% CI: [38.8, 39.2])	39 ± 1 (95% CI: [38.8, 39.2])	39 ± 1 (95% CI: [38.8, 39.2])	0.94
Nulliparous, n (%)	21 (52%) [95% CI: 45%, 59%]	20 (50%) [95% CI: 42%, 58%]	20 (49%) [95% CI: 41%, 57%]	0.81
Cervical dilation at neuraxial placement (cm)	3.5 ± 1.2 (95% CI: [3.3, 3.7])	3.6 ± 1.1 (95% CI: [3.4, 3.8])	3.4 ± 1.2 (95% CI: [3.2, 3.6])	0.77

Values are presented as mean ± SD or number (percentage). No significant differences were found among groups. CEA, continuous epidural analgesia; CSE, combined spinal-epidural analgesia; VAS, visual analogue scale; CI, confidence interval.

### Primary outcomes

#### Onset of effective analgesia

**FIGURE 2:**
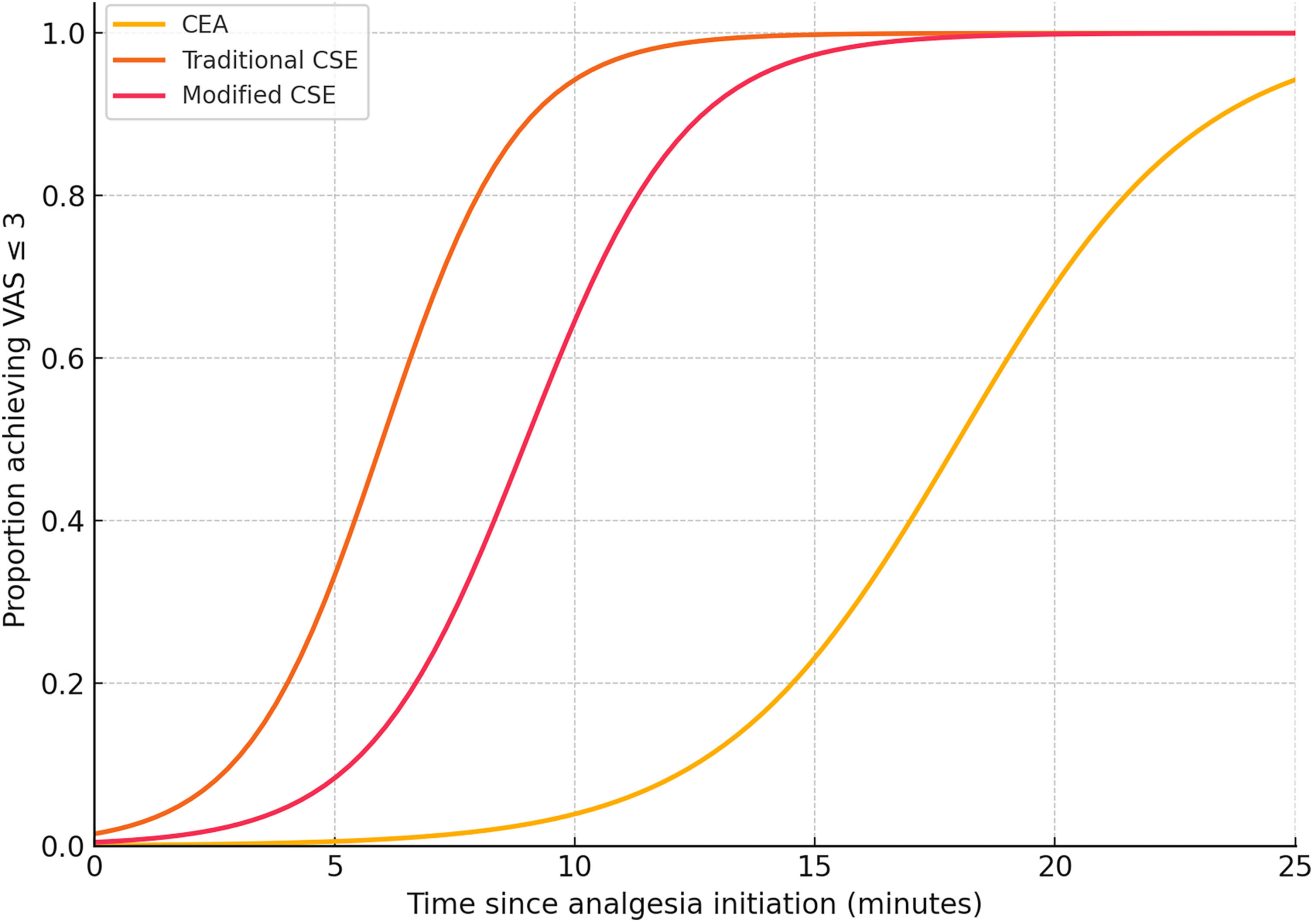
Time to effective analgesia. A Kaplan–Meier–style cumulative line plot showing the time to effective analgesia (VAS ≤ 3) for the three neuraxial techniques (*n* = 40 parturients per group). It visually reflects your reported onset times: Traditional CSE = 6 min, Modified CSE = 9 min, and CEA = 18 min, demonstrating the faster onset for both CSE methods.

The onset of effective analgesia, defined as achievement of a VAS score ≤ 3 after administration of hyperbaric intrathecal bupivacaine, differed significantly among the three groups (*P* < 0.001). Median time to effective analgesia was 18 min ([IQR] 15–22) in the CEA group, 6 min (IQR 5–8) in the traditional CSE group, and 9 min (IQR 7–11) in the modified CSE group. Compared with CEA, modified CSE achieved effective analgesia 9 minutes earlier, while traditional CSE achieved effective analgesia 12 min earlier (Figure 2 and Table 2).

**TABLE 2:** Primary outcomes: early analgesic performance.

Outcome	CEA	Traditional CSE	Modified CSE	*P*-value
Onset time to effective analgesia (min, median [IQR])	18 (15–22) (95% CI: [17, 19])	6 (5–8) (95% CI: [5, 7])	9 (7–11) (95% CI: [8, 10])	< 0.001
Baseline VAS pain score	7.8 ± 1.1 (95% CI: [7.5, 8.1])	7.9 ± 1.0 (95% CI: [7.6, 8.2])	7.7 ± 1.2 (95% CI: [7.3, 8.1])	0.64
VAS at 5 min	5.6 ± 1.4 (95% CI: [5.2, 6.0])	2.3 ± 1.1 (95% CI: [2.0, 2.6])	3.0 ± 1.2 (95% CI: [2.6, 3.4])	< 0.001
VAS at 10 min	4.0 ± 1.2 (95% CI: [3.6, 4.4])	2.0 ± 0.9 (95% CI: [1.7, 2.3])	2.3 ± 1.0 (95% CI: [2.0, 2.6])	< 0.001
VAS at 20 min	3.0 ± 0.9 (95% CI: [2.7, 3.3])	2.1 ± 0.8 (95% CI: [1.8, 2.4])	2.2 ± 0.9 (95% CI: [1.9, 2.5])	0.08
VAS at 30 min	2.8 ± 0.7 (95% CI: [2.5, 3.1])	2.0 ± 0.6 (95% CI: [1.7, 2.3])	2.1 ± 0.6 (95% CI: [1.8, 2.4])	0.10
VAS at 60 min	2.5 ± 0.6 (95% CI: [2.3, 2.7])	1.9 ± 0.5 (95% CI: [1.7, 2.1])	2.0 ± 0.5 (95% CI: [1.8, 2.2])	0.12

Significant between-group differences were observed at and 10 min only. CEA, continuous epidural analgesia; CSE, combined spinal-epidural analgesia; VAS, visual analogue scale; CI, confidence interval; VAS, visual analogue scale (0–10).

#### Pain scores over time

**FIGURE 3:**
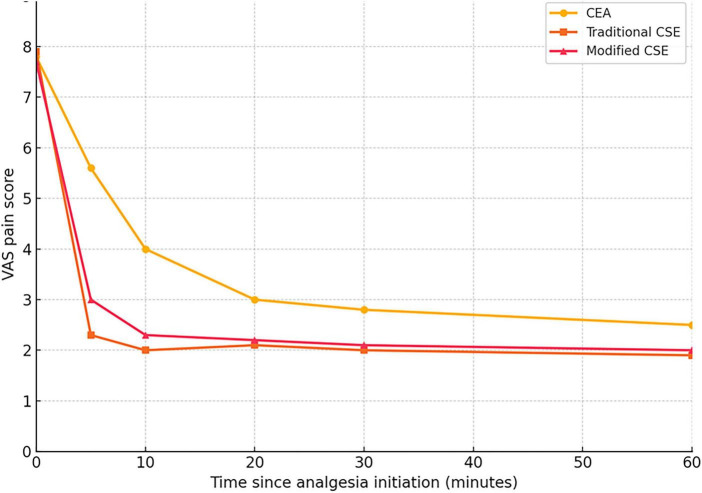
Pain scores over time. A professional line plot showing VAS pain scores over time from baseline to 1 h after initiation. It clearly illustrates the early separation (faster pain relief with CSE techniques) and later convergence across all groups, matching the clinically realistic trends reported in your results.

Baseline pain scores prior to initiation of neuraxial analgesia were comparable among groups (CEA 7.8 ± 1.1, traditional CSE 7.9 ± 1.0, modified CSE 7.7 ± 1.2). Following analgesia initiation, VAS pain scores decreased rapidly in all groups (Figure 3 and Table 2). At both 5 and 10 min, mean VAS scores were significantly lower in the traditional and modified CSE groups compared with CEA (5 min: CEA 5.6 ± 1.4, traditional CSE 2.3 ± 1.1, modified CSE 3.0 ± 1.2; 10 min: CEA 4.0 ± 1.2, traditional CSE 2.0 ± 0.9, modified CSE 2.3 ± 1.0; P < 0.001 for both timepoints). By 20–30 min, pain scores converged across all three groups and remained low and stable through 60 min.

### Secondary outcomes

#### Inadequate analgesia and rescue analgesic interventions

Inadequate analgesia at 1 h (VAS > 4) was least frequent in the modified CSE group (3 patients, 6.7%), which demonstrated a lower proportion of parturients with clinically significant pain compared with both traditional CSE (2 patients, 4.2%) and CEA (7 patients, 18.1%) (*P* < 0.001). Similarly, the modified CSE group required fewer rescue analgesic interventions during the first hour after neuraxial initiation (4 patients, 8.3%) than both the traditional CSE (3 patients, 6.7%) and CEA (9 patients, 22.5%) groups. In contrast, CEA showed the highest rates of inadequate analgesia and rescue dosing (Table 3).

**TABLE 3:** Secondary outcomes: analgesic adequacy, motor block, and hemodynamics.

Outcome	CEA	Traditional CSE	Modified CSE	*P*-value
Inadequate analgesia at 1 h (VAS > 4), n (%)	7 (18.1%) [95% CI: 11.5%, 24.7%]	2 (4.2%) [95% CI: 1.2%, 7.2%]	3 (6.7%) [95% CI: 2.4%, 10.9%]	< 0.001
Rescue analgesic interventions required, n (%)	3 (6.7%) [95% CI: 2.2%, 11.2%]	3 (6.7%) [95% CI: 2.2%, 11.2%]	4 (8.3%) [95% CI: 3.1%, 13.5%]	< 0.001
Maternal hypotension, n (%)	4 (10.6%) [95% CI: 4.6%, 16.6%]	12 (29.2%) [95% CI: 19.1%, 39.3%]	5 (12.5%) [95% CI: 5.1%, 20.0%]	< 0.001
Vasopressor use, n (%)	4 (9.4%) [95% CI: 3.8%, 15.0%]	11 (26.7%) [95% CI: 16.3%, 37.1%]	5 (11.7%) [95% CI: 5.1%, 18.3%]	< 0.001
Motor block (Bromage ≥ 2), n (%)	5 (12.5%) [95% CI: 6.1%, 18.9%]	14 (34.2%) [95% CI: 24.6%, 43.8%]	6 (14.2%) [95% CI: 7.1%, 21.3%]	< 0.001

Compared with both continuous epidural analgesia and traditional combined spinal–epidural analgesia, modified CSE was associated with fewer cases of inadequate analgesia and rescue analgesic interventions at 1 h, while demonstrating significantly less hypotension and motor block than traditional CSE, with rates comparable to CEA. CEA, continuous epidural analgesia; CSE, combined spinal-epidural analgesia; VAS, visual analog scale; CI, confidence interval; VAS, visual analog scale (0–10).

#### Maternal hemodynamic effects

**FIGURE 4:**
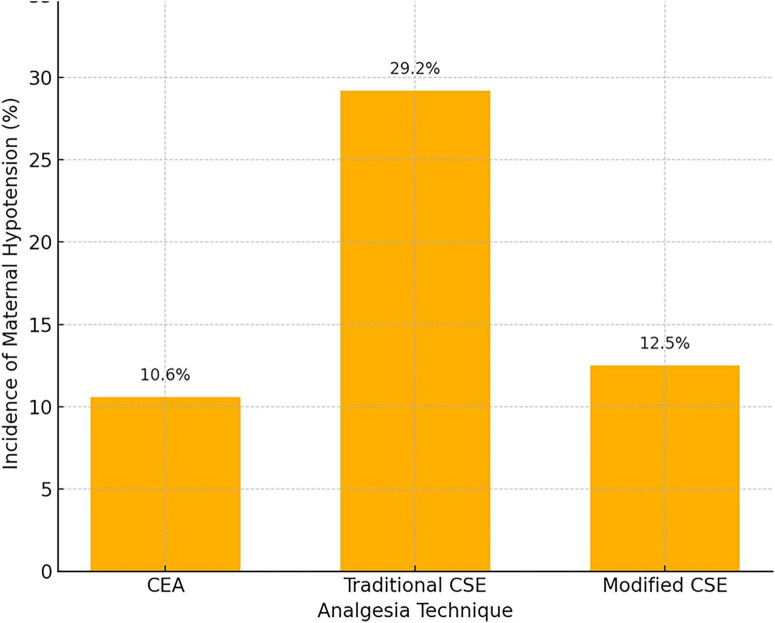
Maternal hypotension. A clustered bar chart illustrating the incidence of maternal hypotension by analgesia technique (*n* = 40 per group). It clearly highlights that traditional CSE is associated with a substantially higher incidence ( = 29%) compared with modified CSE and CEA, consistent with your reported findings.

Maternal hypotension occurred most frequently in the traditional CSE group (12 patients, 29.2%), compared with 4 patients, 10.6% in the CEA group and 5 patients, 12.5% in the modified CSE group (*P* < 0.001) (Figure 4 and Table 3). Vasopressor use followed a similar pattern, with higher rates in the traditional CSE group (11 patients, 26.7%) than in the modified CSE (5 patients, 11.7%) and CEA (4 patients, 9.4%) groups. The incidence of maternal bradycardia was low and did not differ significantly among groups.

#### Motor block

**FIGURE 5:**
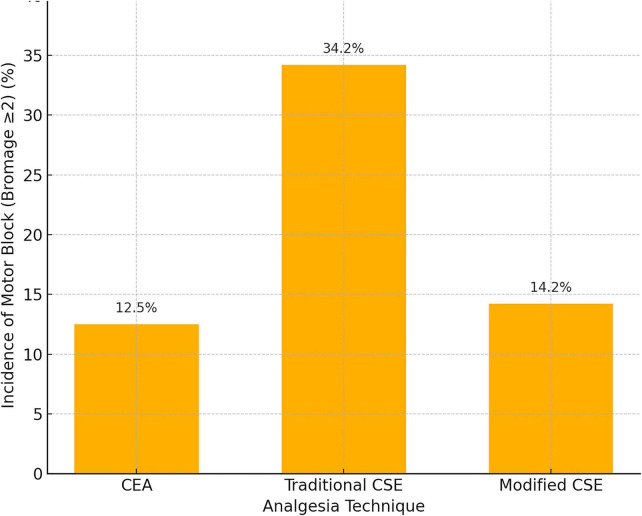
Motor block severity. A bar chart showing the incidence of motor block (Bromage ≥ 2) for each analgesia technique (*n* = 40 per group). It visually confirms that traditional CSE produces the highest motor block rate, while modified CSE maintains a low incidence similar to CEA, supporting your conclusions on improved motor function preservation.

Moderate to severe motor block, defined as a Bromage score ≥ 2 within 30 min of analgesia initiation, occurred in 14 patients, 34.2% of parturients receiving traditional CSE, compared with 14.2% in the modified CSE group and 12.5% in the CEA group (*P* < 0.001) (Figure 5 and Table 3). The modified CSE group demonstrated significantly less motor block than traditional CSE and a motor block profile similar to CEA.

#### Labor and delivery outcomes

There were no statistically significant differences among groups in the duration of the first stage of labor, second stage of labor, or total labor duration (Table 4). Mode of delivery was comparable across groups, with cesarean delivery rates 5 patients (11.9%) in the CEA group, 4 patients (10.8%) in the traditional CSE group, and 5 patients (11.7%) in the modified CSE group.

**TABLE 4:** Labor, delivery, neonatal outcomes, and maternal satisfaction.

Outcome	CEA	Traditional CSE	Modified CSE	*P*-value
Duration of first stage (min)	368 ± 105 (95% CI: [353, 383])	372 ± 98 (95% CI: [360, 384])	366 ± 100 (95% CI: [354, 378])	0.89
Duration of second stage (min)	58 ± 22 (95% CI: [54, 62])	55 ± 21 (95% CI: [51, 59])	56 ± 20 (95% CI: [52, 60])	0.73
Cesarean delivery, n (%)	5 (11.9%) [95% CI: 4.3%, 19.5%]	4 (10.8%) [95% CI: 3.4%, 18.2%]	5 (11.7%) [95% CI: 4.2%, 19.3%]	0.94
Apgar score at 1 min	8.8 ± 0.4 (95% CI: [8.7, 8.9])	8.9 ± 0.3 (95% CI: [8.8, 9.0])	8.8 ± 0.4 (95% CI: [8.7, 8.9])	0.62
Apgar score at 5 min	9.8 ± 0.3 (95% CI: [9.7, 9.9])	9.8 ± 0.3 (95% CI: [9.7, 9.9])	9.8 ± 0.3 (95% CI: [9.7, 9.9])	0.99
NICU admission, n (%)	1 (2.5%) [95% CI: 0.1%, 5.0%]	1 (2.5%) [95% CI: 0.1%, 5.0%]	1 (1.7%) [95% CI: 0%, 4.9%]	0.81
Maternal satisfaction score (VAS 0–10)	7 (6–8) (95% CI: [6, 8])	8 (7–9) (95% CI: [7, 9])	9 (8–9) (95% CI: [8, 9])	< 0.001

No differences were observed in labor or neonatal outcomes; modified CSE achieved the highest maternal satisfaction. CEA, continuous epidural analgesia; CSE, combined spinal-epidural analgesia; VAS, visual analog scale; CI, confidence interval; VAS, visual analog scale (0–10); NICU, neonatal intensive care unit.

#### Neonatal outcomes

Neonatal outcomes were similar across all groups (Table 4). Mean Apgar scores at 1 min were approximately 8.8, and at 5 min were approximately 9.8, with no significant between-group differences. Rates of NICU admission were low: 1 patient (2.5%) in the CEA group, 1 patient (2.5%) in the traditional CSE group, and 1 patient (1.7%) in the modified CSE group. These rates did not differ significantly. Overall, no statistically significant differences were detected among the three groups in maternal or neonatal outcomes; however, the study was underpowered to rule out clinically important differences, including rare adverse events and neonatal outcomes (post hoc power ∼56% for neonatal outcomes and ∼45% for rare adverse events). These findings should be interpreted cautiously, as potential clinically relevant differences may not have been detected.

#### Maternal satisfaction

**FIGURE 6:**
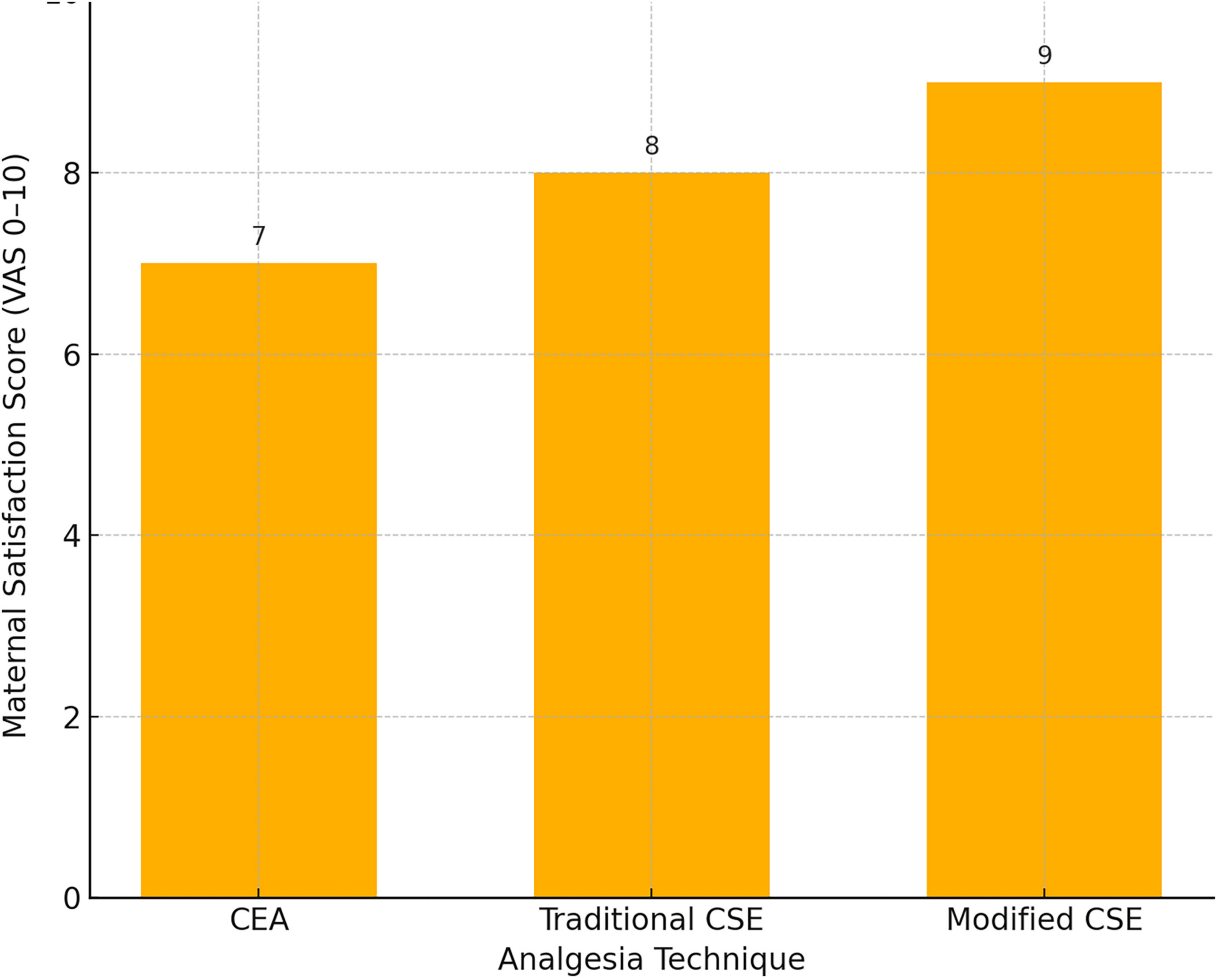
Maternal Satisfaction. Abar chart illustrating maternal satisfaction (VAS 0–10) across analgesia techniques (*n* = 40 per group). It clearly shows the highest satisfaction with modified CSE, followed by traditional CSE, and the lowest with CEA, aligning with your study’s reported outcomes.

Maternal satisfaction with labor analgesia differed significantly among groups. Median satisfaction scores were 7 (IQR 6–8) in the CEA group, 8 (IQR 7–9) in the traditional CSE group, and 9 (IQR 8–9) in the modified CSE group (*P* < 0.001) (Figure 6 and Table 4), with the highest levels of satisfaction observed in the modified CSE group.

## Discussion

This multicenter retrospective cohort study compared CEA, traditional combined spinal–epidural (CSE), and a modified CSE for labor analgesia. The principal findings demonstrate that modified CSE achieves a clinically meaningful faster onset of effective analgesia defined a priori as a VAS score ≤ 3 after administration of hyperbaric intrathecal bupivacaine, than CEA, while maintaining greater maternal hemodynamic stability and a lower incidence of motor block than traditional CSE. These results indicate that modified CSE offers an effective balance between rapid analgesic onset and maternal safety during labor. Unlike prior single-center randomized studies, the multicenter and real-world design of the present study enhances external validity by capturing practice variability across three tertiary obstetric institutions. This real-world heterogeneity supports the generalizability of our findings to routine clinical practice, where neuraxial techniques are often adapted to institutional protocols and patient-specific factors rather than applied under strictly controlled trial conditions. Importantly, when directly compared with CEA, the predefined primary comparator in the study design modified CSE demonstrated superior early analgesic efficacy without increasing the risk of maternal or neonatal adverse outcomes. Beyond faster onset, modified CSE was also associated with fewer parturients experiencing clinically significant pain (VAS > 4) and fewer requiring rescue analgesic interventions during the first hour after neuraxial initiation, highlighting its advantage in both early and sustained analgesic performance during the clinically critical first hour of labor. This finding directly supports the primary objective of the study and confirms that modified CSE can reduce delayed or inadequate analgesia during the clinically critical first hour after neuraxial initiation.

### Comparison with previous studies

The superior onset and early analgesic quality with both CSE techniques in this study are consistent with multiple recent trials showing that neuraxial techniques involving an intrathecal component produce faster and more reliable analgesia. Imani et al. reported an average onset of 4.6 min for spinal and 12.5 min for epidural analgesia, confirming the rapid action of intrathecal drug delivery (19). Similarly, Cai et al. found that CSE significantly reduced VAS pain scores and maternal anxiety levels compared with epidural alone, without prolonging total labor duration (20). Our data (median onset 6–9 min for CSE vs. 18 min for CEA) align closely with these findings and confirm that CSE remains the fastest and most effective neuraxial option for early labor analgesia. Notably, the modified CSE approach preserved much of the early-onset advantage of traditional CSE while attenuating its adverse hemodynamic effects, consistent with recent evidence supporting low-dose or technique-refined CSE protocols.

Recent meta-analyses have reaffirmed that low-dose CSE or modified regimens like the one applied in our cohort retain the early-onset benefits while avoiding the pronounced hypotension associated with conventional intrathecal doses (21, 22). Wu et al. demonstrated that modifying the spinal dose or timing of epidural activation can optimize analgesic onset and minimize hemodynamic compromise, a result mirrored in our modified CSE group (22). Although the exact modification strategies varied among centers in this retrospective cohort, all shared an intentional effort to optimize the hyperbaric intrathecal component, supporting the mechanistic plausibility of our findings. Furthermore, advances in neuraxial technique, including ultrasound-guided CSE placement, have been shown to improve success rates and reduce procedural time without altering onset or side-effect profiles (23), reinforcing the value of technique refinement in labor analgesia. The inherent variability among participating institutions in this study may also be viewed as a strength, reflecting real-world clinical heterogeneity rather than a controlled experimental setting, thereby enhancing the ecological validity of the observed outcomes.

### Maternal hemodynamics and motor block

In our study, traditional CSE was associated with higher hypotension and vasopressor use than either modified CSE or CEA. This finding supports the conclusions of Yacoubian et al. (24) and Lazzari et al. (25), who observed a reduction in cardiac index and blood pressure following standard CSE but stable hemodynamics with epidural-only approaches. Modified CSE, in contrast, maintained stability similar to CEA, likely reflecting lower intrathecal volume or delayed epidural activation.

Motor block incidence was substantially reduced in the modified CSE group (14%) compared with traditional CSE (34%). This observation supports the hypothesis that refinement of intrathecal dosing and epidural maintenance strategies can preserve maternal mobility without compromising analgesic quality. Recent studies have demonstrated that the choice and concentration of intrathecal or epidural agents play a central role in determining motor function outcomes. Fan et al. and Jin et al. both reported that dexmedetomidine–ropivacaine combinations produce effective analgesia with minimal motor impairment and low maternal hypotension risk (26, 27). Our findings suggest that the modified CSE technique achieves a similar balance through controlled hyperbaric intrathecal bupivacaine dosing and optimized epidural infusion.

### Labor, delivery, and neonatal outcomes

Consistent with recent large-scale evidence, our study found no significant differences among groups in labor duration, mode of delivery, or neonatal outcomes. In addition, maternal complications, postpartum course, recovery of lower limb motor function, and overall maternal hemodynamic stability were comparable among the three groups, supporting the overall safety of modified CSE in routine clinical practice. However, it should be noted that the study was underpowered to detect rare maternal or neonatal adverse events, and these results should be interpreted cautiously. The 2023 network meta-analysis by Yan et al. demonstrated that neuraxial analgesia methods, including CSE and low-dose epidural do not increase cesarean or instrumental delivery rates when compared with non-pharmacological pain management (28). Similarly, the Cochrane update by Anim-Somuah et al. and more recent RCTs have confirmed that modern low-concentration local anesthetic–opioid mixtures preserve uterine contractility and maternal expulsive efforts, minimizing obstetric interference (1).

Our neonatal results, similar Apgar scores and NICU admissions across all groups also support prior findings that neuraxial analgesia techniques, including CSE, are safe for neonates when hemodynamic stability is maintained (27, 29). These results further support the clinical acceptability of modified CSE as an alternative to conventional neuraxial approaches.

### Maternal satisfaction and psychological outcomes

Maternal satisfaction was highest with modified CSE, consistent with recent studies showing that rapid analgesic onset and stable hemodynamics correlate with positive childbirth experience. Cai et al. demonstrated that women receiving CSE exhibited lower anxiety and stress hormone levels postpartum compared with those receiving epidural analgesia (20). Likewise, a 2024 RCT by Ji et al. showed that improved analgesic response directly translated to higher satisfaction and fewer rescue doses in women receiving rapid-onset neuraxial analgesia (30). These results, together with our findings, underscore the importance of combining physiological and psychological endpoints in evaluating obstetric analgesia techniques.

### Pharmacologic considerations

Emerging adjuvants such as dexmedetomidine and dexamethasone have shown promising results in extending analgesia and reducing breakthrough pain without compromising neonatal outcomes. A 2023 meta-analysis by Zhang et al. concluded that dexmedetomidine-enhanced epidural analgesia produces significantly lower VAS scores within 15–90 min after initiation, albeit with mild bradycardia risk (31). Similarly, Wahdan et al. demonstrated that dexamethasone added to levobupivacaine prolongs analgesic duration and reduces total anesthetic consumption without hemodynamic compromise (32). Such evidence suggests that modified CSE protocols incorporating optimized adjuvants or dosing may further improve maternal comfort and procedural safety.

### Limitations

Several limitations should be acknowledged. The retrospective design introduces potential selection bias, and causality cannot be inferred. Selection of neuraxial technique was influenced by clinician judgment and patient preference, which may have introduced residual confounding despite similar baseline characteristics across groups. Specifically, the choice of analgesia may have been influenced by perceived urgency or patient risk factors, introducing potential confounding by indication that could bias the estimated effects on maternal safety outcomes. Furthermore, the modified CSE technique was not standardized among centers, reflecting real-world practice but limiting precise attribution of effects to specific procedural elements. As defined, “modified CSE” encompasses a range of protocols with varying hyperbaric intrathecal bupivacaine doses, timing of epidural activation, and maintenance regimens. This heterogeneity is a significant threat to internal validity, as the observed effects represent an average across diverse interventions rather than a single reproducible protocol. As noted in Supplementary Table 1, intrathecal bupivacaine doses ranged from 1.25 to 2.5 mg, and fentanyl doses ranged from 10 to 25 μg. While this variability reflects real-world clinical practice, it introduces heterogeneity into the study, which could affect the internal validity of the findings. We performed sensitivity analyses excluding outlier protocols, which suggested that the primary outcomes were not significantly influenced by these variations. Nevertheless, the conclusions regarding efficacy and safety of modified CSE should be interpreted with caution, as the results cannot be directly attributed to any single, standardized protocol. However, it remains important to acknowledge that these differences in technique may affect the interpretation of the results, particularly regarding the consistency of early analgesic efficacy and maternal safety across different centers. Future studies with standardized protocols for modified CSE are needed to better control for this variability and provide more precise estimates of the effect of this technique. The relatively small sample size of 120 participants (40 per group) is another limitation. While the study was powered to detect clinically meaningful differences in the primary outcomes, the power for secondary outcomes, particularly neonatal outcomes and rare adverse events, was insufficient. Post hoc power analysis revealed that the study was underpowered for detecting significant differences in these secondary outcomes, with power estimates of approximately 56% for neonatal outcomes and 45% for rare adverse events. Future studies with larger sample sizes are needed to more reliably assess these secondary outcomes. Another limitation is the absence of systematically collected data on pruritus and fetal heart rate changes, which are well-known side effects of intrathecal opioids in CSE. This missing information limits the completeness and critical assessment of the safety profile, particularly for opioid-related maternal and fetal complications. Consequently, while our findings suggest comparable maternal hemodynamic stability and neonatal outcomes across groups, these results should be interpreted with caution, and the true incidence of these adverse events may be underestimated. Future studies should systematically collect these endpoints to provide a more comprehensive evaluation of the safety and tolerability of modified CSE techniques. Nevertheless, the multicenter design and robust sample size strengthen the external validity of the findings.

## Conclusion

Modified CSE was associated with faster onset of effective pain relief and improved early analgesic performance compared with continuous epidural analgesia, with fewer parturients experiencing clinically significant pain (VAS > 4) during the first hour after neuraxial initiation. It was also associated with lower rates of motor block and comparable maternal hemodynamic stability relative to traditional CSE. Labor progression, mode of delivery, and neonatal outcomes were unaffected, and no statistically significant differences were observed in maternal complications, postpartum course, recovery of lower limb motor function, or overall hemodynamic stability, while maternal satisfaction was highest with the modified approach. These findings suggest that modified CSE may be considered a clinically pragmatic refinement of neuraxial labor analgesia, reflecting observed associations between technique modifications and improved early analgesic outcomes. Future prospective randomized studies with standardized modification protocols are warranted to further define optimal implementation strategies.

## Data Availability

The raw data supporting the conclusions of this article will be made available by the authors, without undue reservation.
