# Supplementary material for: Comparison of continuous epidural analgesia, traditional combined spinal–epidural, and modified combined spinal–epidural for labor analgesia: a multicenter retrospective cohort study
Source: Front Med (Lausanne). 2026 Jul 8;13:1805555. doi: 10.3389/fmed.2026.1805555 (PMC13388265; doi:10.3389/fmed.2026.1805555)
Supplement: Supplementary file 1 [file Table_1.docx]

**Table S1**. Comparison of Neuraxial Analgesia Techniques and Drug Regimens Across Participating Centers

| **Parameter** | **Continuous Epidural Analgesia (CEA)** | **Traditional Combined Spinal–Epidural (CSE)** | **Modified Combined Spinal–Epidural (Modified CSE)** |
| --- | --- | --- | --- |
| Epidural puncture level | L2–L3 or L3–L4 | L2–L3 or L3–L4 | L2–L3 or L3–L4 |
| Technique | Epidural catheter only | Needle-through-needle CSE | Refined needle-through-needle CSE |
| Intrathecal local anesthetic | None | Hyperbaric bupivacaine 2.5 mg | Hyperbaric bupivacaine 1.25–2.0 mg |
| Intrathecal opioid | None | Fentanyl 15–25 μg | Fentanyl 10–15 μg |
| Rationale for intrathecal dosing | - | Standard rapid-onset dose | Dose reduction to reduce hypotension and motor block |
| Timing of epidural catheter use | Immediate | Immediate | Delayed or low-rate initiation |
| Initial epidural loading dose | Ropivacaine 0.1–0.125%, 10–15 mL, administered immediately after catheter placement | Minimal or omitted; if used, administered within 1–2 min after intrathecal injection | Low-volume or omitted |
| Start of continuous infusion / PCEA | Immediately after loading dose; if loading omitted, started low-volume infusion (e.g., 0.08–0.1% ropivacaine + opioid) immediately | Immediately after intrathecal injection or low-volume infusion started within 1–2 min; PCEA settings per institutional protocol | Lower-concentration infusion and/or modified PCEA |
| Epidural maintenance regimen | Infusion and/or PCEA | Infusion and/or PCEA | Lower-concentration infusion and/or modified PCEA |
| Common maintenance solution | Ropivacaine 0.08–0.1% + opioid | Ropivacaine 0.1% + opioid | Ropivacaine 0.06–0.08% + opioid |
| Targeted clinical effect | Stable, sustained analgesia | Rapid dense analgesia | Rapid onset with improved stability |
| Expected motor block | Minimal | Moderate to higher | Minimal to mild |
| Hemodynamic risk profile | Low | Higher risk of hypotension | Comparable to CEA |
| Center-specific variability | Low | Low | Moderate |
| Interpretive classification | Standard reference | Active comparator | Class of refined CSE techniques |

CEA = continuous epidural analgesia; CSE = traditional combined spinal–epidural; modified CSE = refined CSE techniques using reduced intrathecal doses and/or lower-concentration epidural maintenance to preserve rapid onset while minimizing hypotension and motor block. PCEA = patient-controlled epidural analgesia.
