# Supplementary material for: Comparison of continuous epidural analgesia, traditional combined spinal–epidural, and modified combined spinal–epidural for labor analgesia: a multicenter retrospective cohort study
Source: Front Med (Lausanne). 2026 Jul 8;13:1805555. doi: 10.3389/fmed.2026.1805555 (PMC13388265; doi:10.3389/fmed.2026.1805555)
Supplement: Supplementary file 2 [file Table_2.docx]

**Table S2.** Center-Specific Protocols and Outcomes for Modified CSE

| Center | CEA: Epidural Loading Dose (mL, %) | CSE: Intrathecal Bupivacaine (mg) / Fentanyl (μg) | Modified CSE: Intrathecal Bupivacaine (mg) / Fentanyl (μg) | Time to Effective Analgesia (min) | Maternal Satisfaction Score (0–10) |
| --- | --- | --- | --- | --- | --- |
| Center A | 10–15, 0.1–0.125 | 2.5 / 15 (hyperbaric bupivacaine) | 1.25 / 10 (hyperbaric bupivacaine) | 8 | 9 |
| Center B | 10–15, 0.1–0.125 | 2.5 / 15 (hyperbaric bupivacaine) | 2.0 / 15 (hyperbaric bupivacaine) | 7 | 8 |
| Center C | 10–15, 0.1–0.125 | 2.5 / 15 (hyperbaric bupivacaine) | 2.5 / 25 (hyperbaric bupivacaine) | 9 | 7 |

**Note:** This table displays modified CSE protocols and associated outcomes stratified by center, reflecting variability in hyperbaric intrathecal bupivacaine and fentanyl doses, timing of epidural activation, and maintenance regimens. Subgroup analysis suggests that differences in intrathecal dose and protocol variation did not significantly affect primary outcomes of time to effective analgesia or maternal satisfaction, supporting the robustness of the overall results despite heterogeneity in practice.
